# Targeting mTOR/p70S6K/glycolysis signaling pathway restores glucocorticoid sensitivity to 4E-BP1 null Burkitt Lymphoma

**DOI:** 10.1186/s12885-015-1535-z

**Published:** 2015-07-20

**Authors:** Ling Gu, Liping Xie, Chuan Zuo, Zhigui Ma, Yanle Zhang, Yiping Zhu, Ju Gao

**Affiliations:** 1 Laboratory of Hematology/Oncology, Department of Pediatric Hematology/ Oncology, Key Laboratory of Birth Defects and Related Diseases of Women and Children (Ministry of Education), West China Second University Hospital, Sichuan University, Chengdu, 610041 China; 2 Department of Hematology, West China University Hospital, Sichuan University, Chengdu, 610041 China; 3 Department of Rheumatology, West China University Hospital, Sichuan University, Chengdu, 610041 China

**Keywords:** Rapamycin, Mammalian target of rapamycin, p70S6 kinase, Glucocorticoid, Resistance, Raji, Burkitt lymphoma, Glycolysis

## Abstract

**Background:**

Increasing evidence indicates that rapamycin could be used as a potential glucocorticoid (GC) sensitizer in lymphoblastic malignancies via genetic prevention of 4E-BP1 phosphorylation. Interestingly, we found that combined rapamycin with dexamethasone can effectively reverse GC resistance in 4E-BP1 null lymphoma cells. In this study, we investigated the potential link between mTOR/p70S6K signaling pathway, glycolysis, autophagy and GC resistance.

**Methods:**

Antitumor effects of the combination of rapamycin and dexamethasone were evaluated on cell viability by MTT assay and in vivo studies, on cell cycle and apoptosis by flow cytometry, on autophagy by western blot, MDC staining and transmission electron microscopy and on cell signaling by western blot. Moreover, to test whether inhibiting glycolysis is the core mechanism in rapamycin restoring GC sensitivity, we took glycolysis inhibitor 2-deoxyglucose to replace rapamycin and then evaluated the antitumor effects *in vitro*.

**Results:**

Raji cells are resistant to rapamycin (IC_50_ > 1000 nM) or dexamethasone (IC_50_ > 100 μM) treatment alone. The combination of rapamycin and dexamethasone synergistically inhibited the viability of Raji cells *in vitro* and *in vivo* by inducing caspase-dependent and -independent cell death and G_0_/G_1_ cell cycle arrest. These effects were achieved by the inhibition of mTOR/p70S6K signaling pathway, which led to the inhibition of glycolysis and the induction of autophagy. Pretreatment with pan-caspase inhibitor z-VAD-fmk or autophagy inhibitor 3-MA failed to protect the cells from combined treatment-induced death. Glycolysis inhibitor combined with dexamethasone produced a similar antitumor effects *in vitro.*

**Conclusions:**

Inhibition of mTOR/p70S6K/glycolysis signaling pathway is the key point of therapy in reversing GC resistant in Burkitt lymphoma patients.

**Electronic supplementary material:**

The online version of this article (doi:10.1186/s12885-015-1535-z) contains supplementary material, which is available to authorized users.

## Background

Glucocorticoids (GCs) induce cell cycle arrest and apoptosis in lymphoblastic cells and therefore constitute a central component in the treatment of lymphoid malignancies. GC resistance is a therapeutic problem with an unclear molecular mechanism [1]. We have demonstrated that rapamycin (Rap), a mammalian target of rapamycin (mTOR) inhibitor, can effectively sensitize anaplastic lymphoma kinase-positive lymphoid cells to dexamethasone (Dex)-induced apoptosis [2]. Rap could be used as a potential GC sensitizer in hematological malignancies [3–7]. mTOR is a serine-threonine protein kinase that belongs to the phosphoinositide 3-kinase (PI3K)-related kinase family. The inhibition of mTOR kinase leads to dephosphorylation of its two major downstream signaling components, p70S6 kinase (p70S6K), a kinase implicated in cell proliferation, and eukaryotic initiation factor 4E binding protein 1 (4E-BP1), a protein that inhibits the translation of 5’-cap mRNAs [8]. A previous study has reported that genetic prevention of 4E-BP1 phosphorylation (p-4E-BP1) but not p70S6K phosphorylation (p-p70S6K) enhances Dex-induced apoptosis in multiple myeloma cells [4]. In addition, 4E-BP1 expression correlates with resistance to mTOR inhibitors [9, 10].

The GC-resistant Raji cell line, established in 1963 from the left maxilla of a 12-year-old African boy with Burkitt lymphoma [11], with 4E-BP1-null [12], t(8;14), and high c-Myc expression, is a Rap-resistant cell line [9, 13]. Unexpectedly, our data showed that Rap effectively potentiates Dex-induced apoptosis in the 4E-BP1-null Raji cells. There should have other underlying mechanisms for the association between mTOR activation and GC resistance.

An increasing number of studies have reported that increased aerobic glycolysis is a hallmark of cancer and plays a role in the chemoresistance of different tumor cells [14–16]. Interestingly, in addition to being a key mediator that regulates cell survival, S6K is also a critical mediator of glycolytic metabolism in mTOR-activated cells [17]. Targeting glycolysis sensitizes tumor cells to chemotherapy [18, 19]. Inhibition of the mTOR pathway sensitizes leukemia cells to aurora inhibitors by suppression of the glycolytic metabolism [20]. More interestingly, mTOR is also a master negative regulator of autophagy [21]. Bonapace [22] reported that induction of autophagy-dependent necroptosis is required for childhood acute lymphoblastic leukemia cells to overcome GC resistance. There should have potential links among the mTOR/p70S6K signaling pathway, glycolysis, autophagy and GC resistance.

In our current study, we have shown that the combination of Rap with Dex effectively inhibited the mTOR/p70S6K/glycolysis signaling pathway and induced autophagy, which led to the restoration of GC sensitivity in Rap- and Dex-resistant Raji cells *in vitro* and *in vivo.* Therefore, the combination of an mTOR inhibitor with Dex is a promising therapeutic approach for GC-resistant Burkitt lymphoma. More importantly, the study provides further insight into the molecular mechanisms involved in Rap reversing GC resistance. Components of mTOR/p70S6K/glycolysis signaling network could be targeted for the reversion of GC resistance.

## Methods

### Cell line and culture conditions

The Burkitt lymphoma cell line Raji was purchased from the Shanghai Institute Cell Resources Bank. Raji cells were maintained in RPMI 1640 (Hyclone, Logan, USA) supplemented with 10 % fetal bovine serum, 2 mM L-glutamine (Hyclone) and antibiotics (100 U/ml penicillin and 50 μg/ml streptomycin) at 37 °C in a humidified 5 % CO_2_ in-air atmosphere.

### Reagents and antibodies

As described previously [2], Rap (Calbiochem, San Diego, CA, USA) was dissolved in dimethyl sulfoxide (DMSO, Sigma, St. Louis, MO, USA) and used at a concentration of 10 nM. Dex (Sigma) was dissolved in ethanol and used at a concentration of 1 μM. The final concentrations of DMSO and ethanol in the medium were 0.05 % and 0.01 %, respectively, at which cell proliferation or viability was not obviously altered. Propidium iodide (PI), 3-methyladenine (3-MA), 2-deoxyglucose (2-DG) and 3-(4,5-dimethylthiazol-2-yl)-2,5-diphenyltetrazolium bromide (MTT) were purchased from Sigma. The pan-caspase inhibitor z-VAD-fmk was purchased from R&D Systems (Minneapolis, MN, USA). The Annexin V-PI Kit was purchased from Roche (Mannheim, Germany). Antibodies to phospho-glucocorticoid receptor (p-GR) (Ser211), p70S6K, p-p70S6K (Thr421/Ser424), 4E-BP1, p-4E-BP1 (Thr37/46), AMP-activated protein kinase (AMPK), phospho-AMPK (p-AMPK) (Thr172), Cyclin D, p27, Bax, Mcl-1, and Bcl-2 were purchased from Cell Signaling Technology (Beverly, MA, USA). The antibody for p21 was purchased from BD Bioscience (San Jose, CA, USA). Antibodies to extracellular signal-regulated kinase (ERK) and phospho-ERK (p-ERK) were purchased from Upstate/Millipore (Billerica, MA, USA). Antibody to LC3 was purchased from Sigma. Antibodies to GR, Bim, Cyclin A, horseradish peroxidase (HRP)–conjugated donkey anti-rabbit antibody and HRP-conjugated sheep anti-mouse antibodies were obtained from Santa Cruz Biotech (Santa Cruz, CA, USA). The actin antibody was obtained from Kangchen Bio-Tech (Shanghai, China).

### Cell treatment

Logarithmically growing cells were harvested and plated in 96-well sterile plastic culture plates and 25-cm^2^ flasks (Corning Inc.), to which various concentrations of Rap or Dex, specifically 10 nM Rap (Rap group), 1 μM Dex (Dex group), 10 nM Rap plus 1 μM Dex (Rap + Dex group) and 0.05 % DMSO plus 0.01 % ethanol (Control group), were added. At the end of the incubation period, cells were transferred to sterile centrifuge tubes, pelleted by centrifugation at 400 *g* at room temperature for 5 min, and prepared for analysis as described below.

### Cell viability assay

MTT assays were performed as described previously. Briefly, cells were seeded in 96-well plates (100,000/ml) and incubated for 24 or 48 h. Next, 0.5 mg/ml MTT (final concentration) was added to each well for 4 h at 37 °C. Then, solubilization buffer (10 % SDS in 0.01 M HCl) was added to each well, and the plates were further incubated for 24 h at 37 °C. The spectrophotometric absorbance was measured at 570 nm (reference 690 nm) using a multi-plate reader (Multiskan Spectrum, Thermo Electron Co., Waltham, MA, USA). Values were obtained by comparing the experimental cells with their respective controls. Mean values were calculated from triplicate cultures. Coefficient of drug interaction (CDI) was used to analyze the effects of drug combinations. The CDI is calculated as follows: CDI = AB/(A × B). According to the absorbance of each group, AB is the ratio of the combination groups to control group; A or B is the ratio of the single agent group to control group. Thus, a CDI value <1, =1 or >1 indicates that the drugs are synergistic, additive or antagonistic, respectively.

### Cell cycle analysis

For each analysis, 10^6^ cells were harvested 48 h after treatment and fixed overnight in 70 % ethanol at 4 °C. Cells were then washed and stained with 5 μg/ml PI in the presence of DNAse-free RNAse (Sigma). After 30 min at room temperature, the cells were analyzed via flow cytometry (Beckman Coulter Inc., Miami, FL, USA), acquiring 30,000 events.

### Apoptosis assay

The samples were washed with phosphate-buffered saline (PBS) twice and stained with annexin V-FLUOS and PI using Annexin-V-FLUOS staining kit (Roche) according to the manufacturer protocol. The percentages of annexin-V single positive cells were determined by flow cytometry (Beckman Coulter), as the percentages of cells in the early stages of apoptosis.

### Glucose consumption assay

Glucose consumption was measured with a Glucose (HK) Assay Kit (Sigma). Briefly, 1 × 10^6^ cells were grown in 10 ml RPMI containing 2 g/l glucose. After 48 h, the medium was collected by centrifugation to remove the cells. Medium from each condition was incubated for 30 min with the glucose assay reagent. Spectrophotometric absorbance was measured at 340 nm using a multi-plate reader (Multiskan Spectrum). Values were obtained by comparing with a glucose standard solution.

### Lactic acid assay

Lactic acid production was measured with a Lactic Acid Assay Kit (Jiancheng, Nanjing, China). Briefly, 1 × 10^6^ cells were grown in 10 ml RPMI. After 48 h, the medium was collected by centrifugation to remove the cells. Medium from each condition was incubated with the lactic acid assay reagent according to the manufacturer protocol. Spectrophotometric absorbance was measured at 530 nm using a multi-plate reader (Multiskan Spectrum). Values were obtained by comparing with a lactic acid standard solution.

### In vivo studies

All animal studies were conducted in accordance with the guidelines established by the internal Institutional Animal Care and Use Committee and Ethics Committee guidelines of Sichuan University. All animals were kept under specific pathogen-free conditions in Laboratory Center of West China Second Hospital, Sichuan University. Female Balb/c (nu/nu) mice (Laboratory Animal Center of Sichuan University, Chengdu, China), 5–6 weeks of age, 16-18 g of weight, were inoculated with 3 × 10^6^ Raji cells subcutaneously (s.c.) in the right flank with an inoculation volume of 0.2 ml. Tumor size was measured by calipers every 2 days. The approximate tumor volume was calculated using the equation V = (length × width × depth)/2. Once palpable tumors were established (tumor volume reaching 30–40 mm^3^), animals were randomized into 4 groups, each containing 6 mice. Mice were injected intraperitoneally daily with 3 mg/kg/d Rap (Rap group), 15 mg/kg/d Dex (Dex group), 3 mg/kg/d Rap plus 15 mg/kg/d Dex (Rap + Dex group) or PBS (Control group). All animals were ear-tagged and monitored individually throughout the experiment.

### Mitochondrial membrane potential detection

The mitochondrial membrane potential (∆ψm) was measured using Rhodamine 123 (Rh123) staining. In brief, Rh123 (10 μM) was loaded into cells for 20 min at 37 °C. The fluorescence intensity of cells was analyzed by flow cytometry (Beckman Coulter) with an excitation wavelength at 488 nm and an emission wavelength at 525 nm.

### Analysis of autophagy with MDC staining

The cells were suspended in 0.05 mM Monodansylcadaverine (MDC, Sigma) and incubated at 37 °C for 40 min. Then, the fluorescent changes were observed by fluorescence microscopy (Olympus, Tokyo, Japan) with the emission wavelength at 525 nm.

### Transmission electron microscopy

Cells were harvested after treatment. Following fixation in 2 % paraformaldehyde/2.5 % glutaraldehyde, pellets were rinsed and post-fixed in 1 % osmium tetroxide/1.25 % potassium ferrocyanide. Samples were dehydrated in a graded series of ethanol, followed by propylene oxide and infiltrated and embedded in Polybed 812 resin. Ultrathin 70-nm-thick sections were taken from areas selected by light microscopy, mounted on 200 mesh copper grids, and stained with uranyl acetate and lead citrate. These were observed and photographed using a Jeol J EM-1200EX transmission electron microscope (Jeol Ltd., Tokyo, Japan).

### Western blotting analysis

 Cells (10^6^) were washed twice in cold PBS and then lysed by Laemmli sample buffer (Bio-Rad). Samples were boiled for 5 min at 100 °C. Proteins were separated by 10 % or 15 % SDS–polyacrylamide gel electrophoresis and transferred onto nitrocellulose membranes (0.22 μm or 0.45 μm, Millipore). Non-specific binding sites were blocked with 5 % non-fat dry milk dissolved in TBS (10 mM Tris–HCl, pH 7.6, 137 mM NaCl) with 0.1 % Tween 20 (TTBS) for 2 h at room temperature, followed by incubation with primary antibody for 2 h at room temperature or at 4 °C overnight. The membranes were then washed 3 times in TTBS and incubated for 2 h at room temperature with secondary HRP–conjugated donkey anti-rabbit antibody or HRP-conjugated sheep anti-mouse antibody (Santa Cruz) diluted 1:5000 in TTBS with 5 % non-fat milk. Proteins were visualized by incubation with ECL plus (Millipore). All experiments were carried out independently at least 3 times. The level of Actin protein was used as a control for the amount of protein loaded into each lane.

### Statistical analysis

All assays were performed in triplicate, and data are expressed as mean values ± SD. One-way ANOVA was used to compare two groups. A *p*-value < 0.05 was considered to be significant.

## Results

### Raji cells are resistant to Rap or Dex treatment alone

**Fig. 1:**
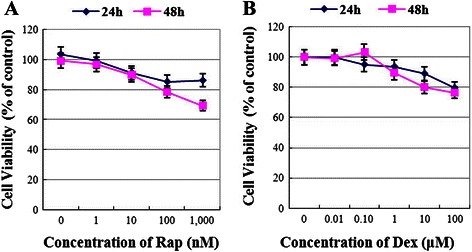
Raji cells are resistant to Rap or Dex treatment alone. **a** Raji cells were cultured with various concentrations of Rap (ranging from 0.1 to 1000 nM) for 24 h and 48 h. The viability rates of the cells were evaluated with an MTT assay. The experiments were performed in triplicate. **b** Raji cells were cultured with various concentrations of Dex (ranging from 0.01 to 100 μM) for 24 h and 48 h. The viability rates of the cells were evaluated with an MTT assay. The experiments were performed in triplicate

Raji cells were treated with various concentrations of Rap or Dex alone for 48 h, followed by assessment of cell viability using MTT assays. No significant concentration-dependent decrease in cell viability was observed in response to Rap or Dex treatment (Fig. 1a and b). After 48 h treatment, Rap inhibited the viability of Raji cells slightly at a regular concentration (10 nM); when the concentration was increased to 1000 nM, the cells exhibited a 30 % viability inhibition (Fig. 1a). Thus, the IC_50_ (concentration that inhibits 50 %) of Rap is higher than 1000 nM in Raji cells. Additionally, 1 μM Dex alone showed almost no effect on cell viability. When the concentration was increased to 100 μM, the cell line exhibited a 25 % viability inhibition at 48 h (Fig. 1b).

### The combination of Rap with Dex effectively inhibits the growth of Rap- and Dex-resistant Raji cells in vitro and in vivo

**Fig. 2:**
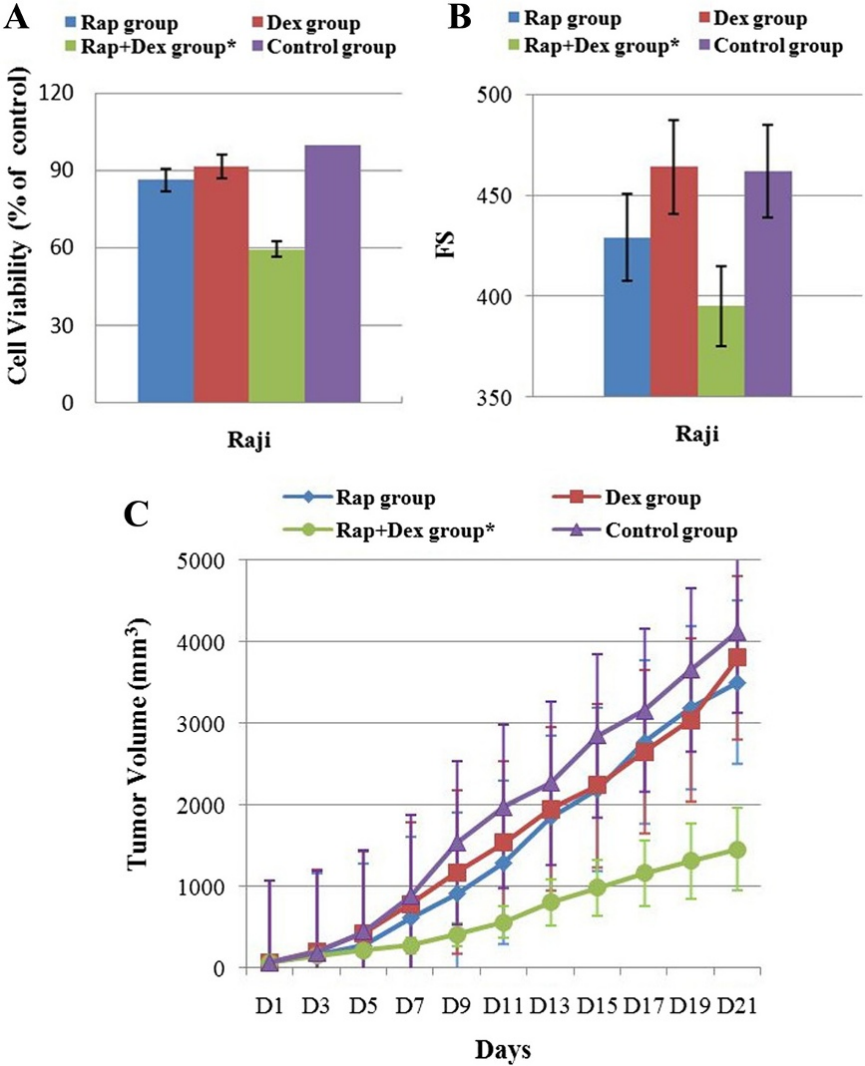
The combination of Rap with Dex effectively inhibits the growth of Rap- and Dex-resistant Raji cells in vitro and in vivo. **a** Raji cells were incubated with Rap (10 nM) and/or Dex (1 μM) for 48 h. The viability of the cells were evaluated with an MTT assay. **b** Flow cytometric analysis showed the cell size (as seen by FS) after 48 h treatment with 10 nM Rap and/or 1 μM Dex. **c** Combined treatment significantly inhibited tumor growth in Raji cell xenografts in nude mice (*n* = 6 per group, age 5 ~ 6 weeks, average-weight 16.9 ~ 17.1 g). All animal procedures were carried out in accordance with ARRIVE guidelines (Additional file 1). *: *p* < 0.01 versus the control group, Dex group, or Rap group

We incubated Raji cells with 10 nM Rap and/or 1 μM Dex for 48 h. Rap alone induced an approximately 13 % reduction in cell viability, and Dex alone induced an approximately 9 % reduction in cell viability. However, when provided in combination, Rap and Dex achieved more than a 40 % cell reduction (Fig. 2a). Rap and Dex combination treatment inhibited viability of Raji cells synergistically, with a CDI of 0.75 ± 0.04. Using a light microscope, we found that the cell size decreased and that cell aggregation was obviously reduced in the Rap + Dex group. Flow cytometric analysis showed that 48 h treatment with 10 nM Rap clearly reduced cell size as seen by the leftward shift of the mean forward scatter (FS), but combining Rap with 1 μM Dex made the cell size smaller and Dex alone did not affect the cell size (Fig. 2b).

Having shown that combined treatment induced cell viability inhibition in Raji cells *in vitro*, we examined the *in vivo* efficacy of the two drugs given intraperitoneally in Raji xenografts in nude mice. As shown in Fig. 2c, 3 mg/kg/d Rap or 15 mg/kg/d Dex used alone showed almost no antitumor effect, whereas combined treatment significantly inhibited tumor growth when compared to the Rap, Dex, and control groups (*P* < 0.001).

### Combination of Rap with Dex arrests Raji cells in G_0_/G_1_ phase of the cell cycle

**Fig. 3:**
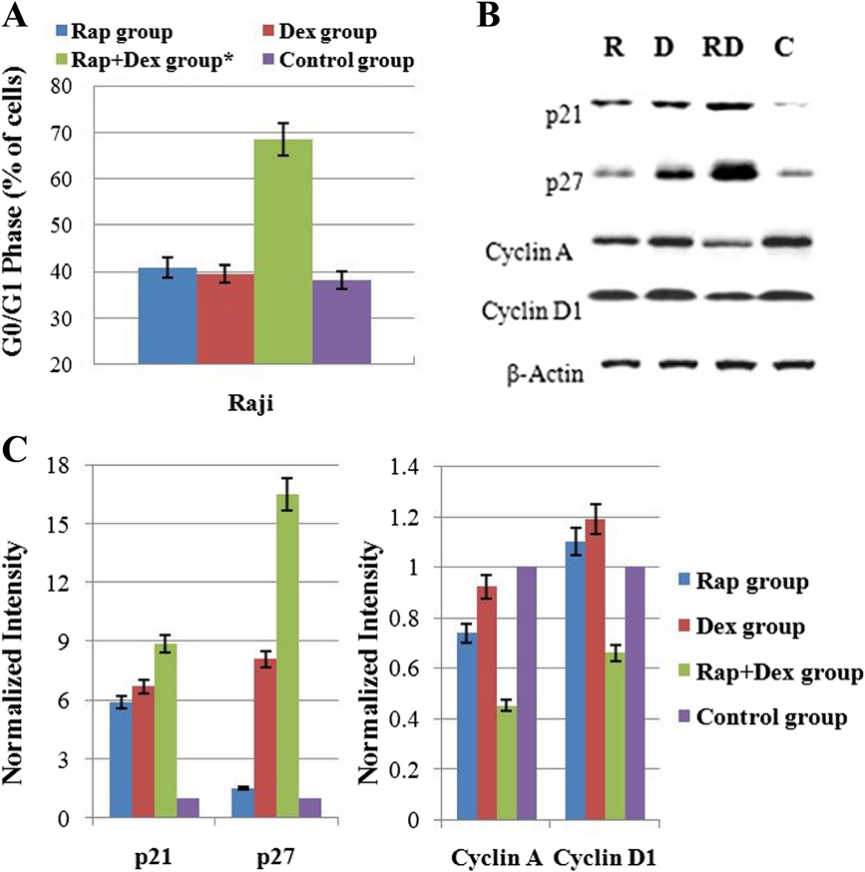
The combination of Rap with Dex arrests Raji cells in the G_0_/G_1_ phase of the cell cycle. **a** Raji cells were incubated for 48 h with Rap (10 nM) and/or Dex (1 μM), and the cell cycle progression was analyzed by PI staining. For all experiments, values are presented as the mean ± SD (*n* = 3) *: *p* < 0.01 versus the control group, Dex group, or Rap group. **b** After 48 h exposure to Rap and/or Dex, cells were lysed and extracts were analyzed by western blotting. β-Actin was used as an internal control. **c** Bar graphs show the normalized intensity of the different proteins. Values are the results of 3 determinations. R, Rap; D, Dex; RD, Rap + Dex, and C, control

Rap, at regular dosages, inhibits cell growth of hematological malignancies by inducing a G_0_/G_1_ arrest without inducing apoptosis [2, 4, 8]. Dex inhibits tumor cell growth mainly by inducing apoptosis. Flow cytometric analysis showed that 48 h treatment with 10 nM Rap or 1 μM Dex alone did not induce G_0_/G_1_ arrest in Raji cells. Interestingly, combined treatment clearly induced G_0_/G_1_ arrest (Fig. 3a). To evaluate the molecular basis underlying the cell cycle arrest, we investigated the expression of cell cycle regulatory proteins. As shown in Fig. 3b and c, after 48 h treatment, combined treatment induced the expression of cyclin-dependent kinase (CDK) inhibitors of p21 and p27 (especially p27) and reduced Cyclin A and Cyclin D1 levels.

### Rap sensitizes Raji cells to Dex-induced apoptosis

**Fig. 4:**
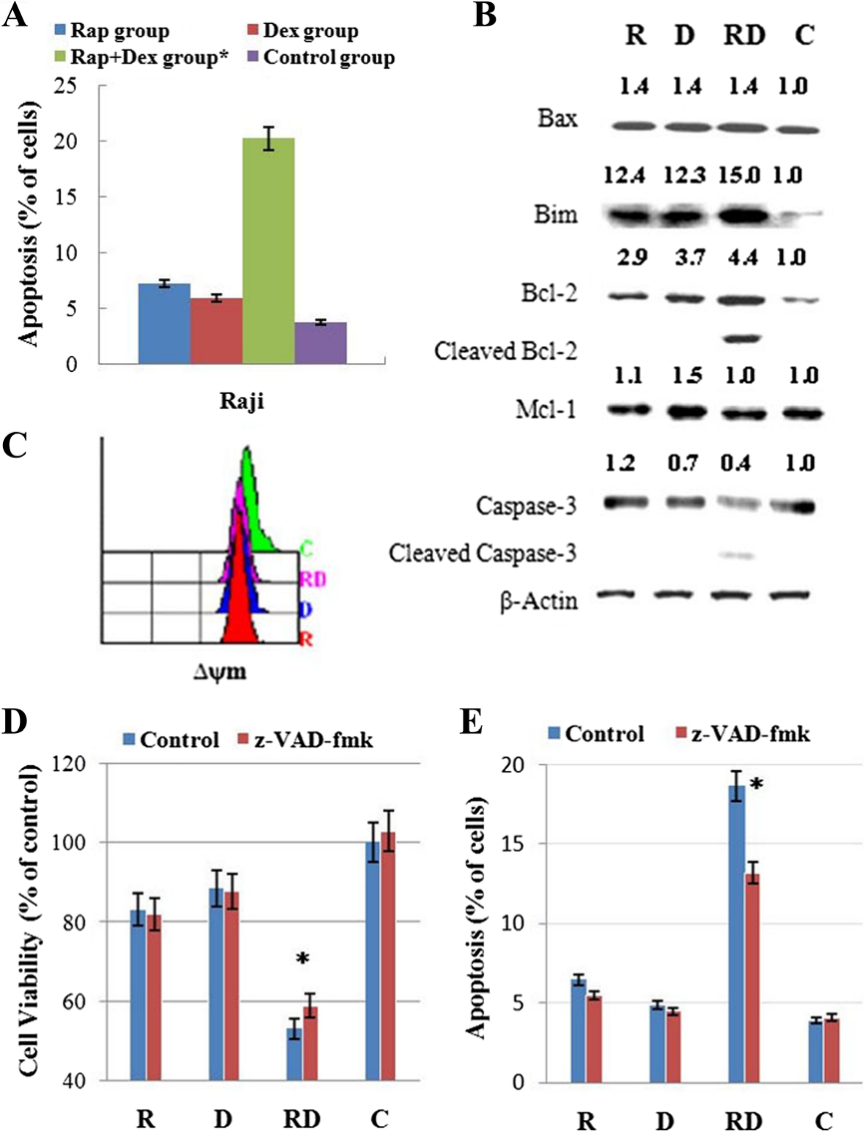
Rap treatment sensitizes Raji cells to GC treatment by inducing apoptosis. **a** Raji cells were incubated for 48 h with Rap (10 nM) and/or Dex (1 μM), and the early stage of apoptosis was detected by Annexin V-FLUOS/PI staining (Annexin V-FLUOS positive/PI negative). For all experiments, values of triplicate experiments are shown as the mean ± SD; *: *p* < 0.01 versus the control group or Dex group or Rap group. **b** After 48 h exposure to Rap and/or Dex, cells were lysed and extracts were analyzed by western blotting for Bcl-2 family proteins and caspase-3. The experiments were performed in triplicate. β-Actin was used as an internal control. **c** ∆ψm was detected by Rh123 staining. **d** The cells were pretreated with 20 μM z-VAD-fmk or 0.1 % DMSO as a control for 2 h in four groups. The viability rates of the cells were evaluated by MTT assays. **e** The early stage of apoptosis was detected by Annexin V-FLUOS/PI staining. *: *p* < 0.05 versus the control in the RD group. R, Rap; D, Dex; RD, Rap + Dex and C, control

The main mechanism of Dex in the treatment of lymphoid malignancies is to induce apoptotic cell death. We used Annexin V-PI staining to determine the early stage of apoptosis. Single treatment with 1 μM Dex or 10 nM Rap had no apoptotic effect on Raji cells; however, when used in combination, a remarkable increase in cell apoptosis was observed (Fig. 4a). Therefore, Rap can effectively sensitize Raji cells to Dex-induced apoptosis. Bcl-2 family members play an important role in GC-induced apoptosis [23]. We then examined the expression of Bcl-2, Bax, Bim-EL, Mcl-1 and caspase-3. Bim was clearly induced in Rap, Dex, and Rap + Dex group, Bax was elevated slightly in the three group, Mcl-1 was induced in Dex group only, Bcl-2 and caspase-3 was cleaved only in combined treatment group (Fig. 4b). These data support that, at least in part, Rap reverses GC resistance via activation of the intrinsic apoptotic program. Next, we analyzed the changes in ∆ψm. As shown in Fig. 4c, Rap and Dex alone or in combination dissipated ∆ψm, and there were no significant differences between them. To determine whether the apoptosis triggered by Rap and Dex was caspase-dependent or caspase-independent, the cells were pretreated with the pan-caspase inhibitor z-VAD-fmk. The cell viability did not change in response to Rap or Dex in cells pre-treated with 20 μM z-VAD-fmk but was induced slightly in the Rap + Dex group compared with the control group (p < 0.05) (Fig. 4d). And the cell apoptosis rate did not change in response to Rap or Dex pre-treatment with 20 μM z-VAD-fmk but reduced in the Rap + Dex group compared with the control group (p < 0.05) (Fig. 4e). Pretreatment with z-VAD-fmk failed to fully protect Raji cells from apoptosis and cell death. These findings suggest that combined treatment induces cell death through both caspase-dependent and caspase-independent mechanisms in Raji cells.

### Combination of Rap with Dex induces autophagic cell death

**Fig. 5:**
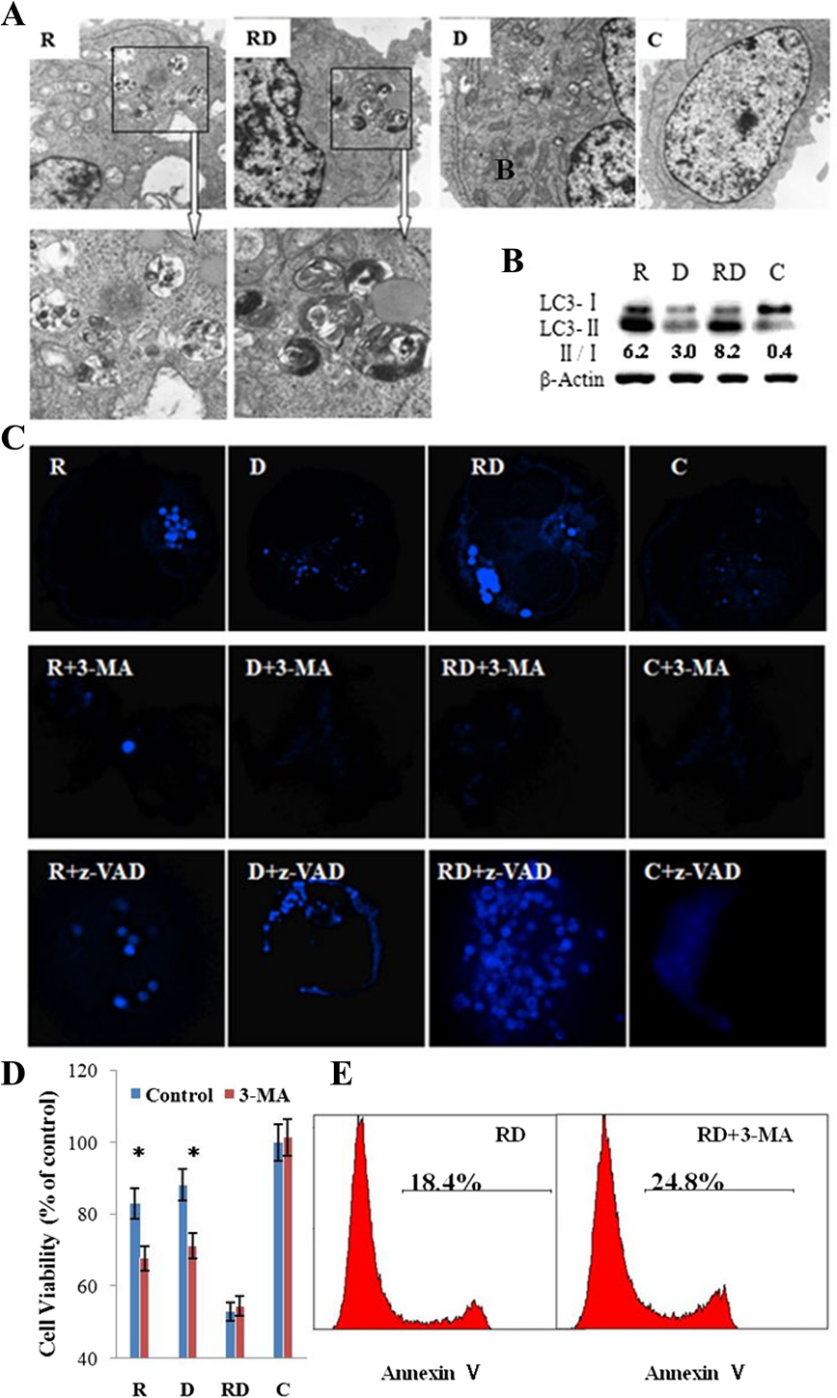
The combination of Rap with Dex induces autophagic cell death. **a** After 24 h exposure to Rap and/or Dex, ultrastructural changes were examined by transmission electron microscopy. Arrows indicate the autophagic vacuoles in the R and RD groups (8000× magnification). **b** Cells were lysed and extracts were analyzed by western blotting for LC3. The experiments were repeated three times and the data show the representative results. **c** MDC staining revealed the formation of autophagosomes (1000× magnification). **d** The cells were pretreated with 1 mM 3-MA or 0.1 % DMSO as a control for 2 h in four groups. The viability rates of the cells were evaluated by MTT assays. *: *p* < 0.05 versus control in the R and D groups. **e** Apoptosis was detected by Annexin V-FLUOS/PI staining (Annexin V-FLUOS positive/PI negative). R, Rap; D, Dex; RD, Rap + Dex and C, control

By far, the potentially most-studied of the caspase-independent cell death mechanisms is autophagic cell death [24]. Rap is known to be an inducer of autophagy. Although Raji cells are resistant to Rap treatment, Rap alone induced the formation of autophagosomes and the generation of LC3-II, Dex alone slightly induced autophagy, and the combined treatment strongly induced autophagy (Fig. 5a, b and c). Because autophagy can result in both cell survival and death, we next determined whether Rap- and Dex-induced autophagy is protective. Pre-incubation with the autophagy inhibitor 3-MA abolished autophagosome formation (Fig. 5c) and reduced the cell viability in Rap and Dex alone groups (Fig. 5d). Therefore, autophagy promoted survival in the cells treated with Rap or Dex alone. However, in the combined group, inhibiting autophagy did not affect the cell viability by inducing apoptosis (Fig. 5d and e). Similarly, in the combined group, when caspase-dependent apoptosis was blocked, autophagy was strongly induced (Fig. 5c). These findings implicate autophagy as a part of a cell death mechanism for GC resensitization by Rap.

### The combination of Rap with Dex acts synergistically on the dephosphorylation of p70S6K and inhibition of glycolysis

**Fig. 6:**
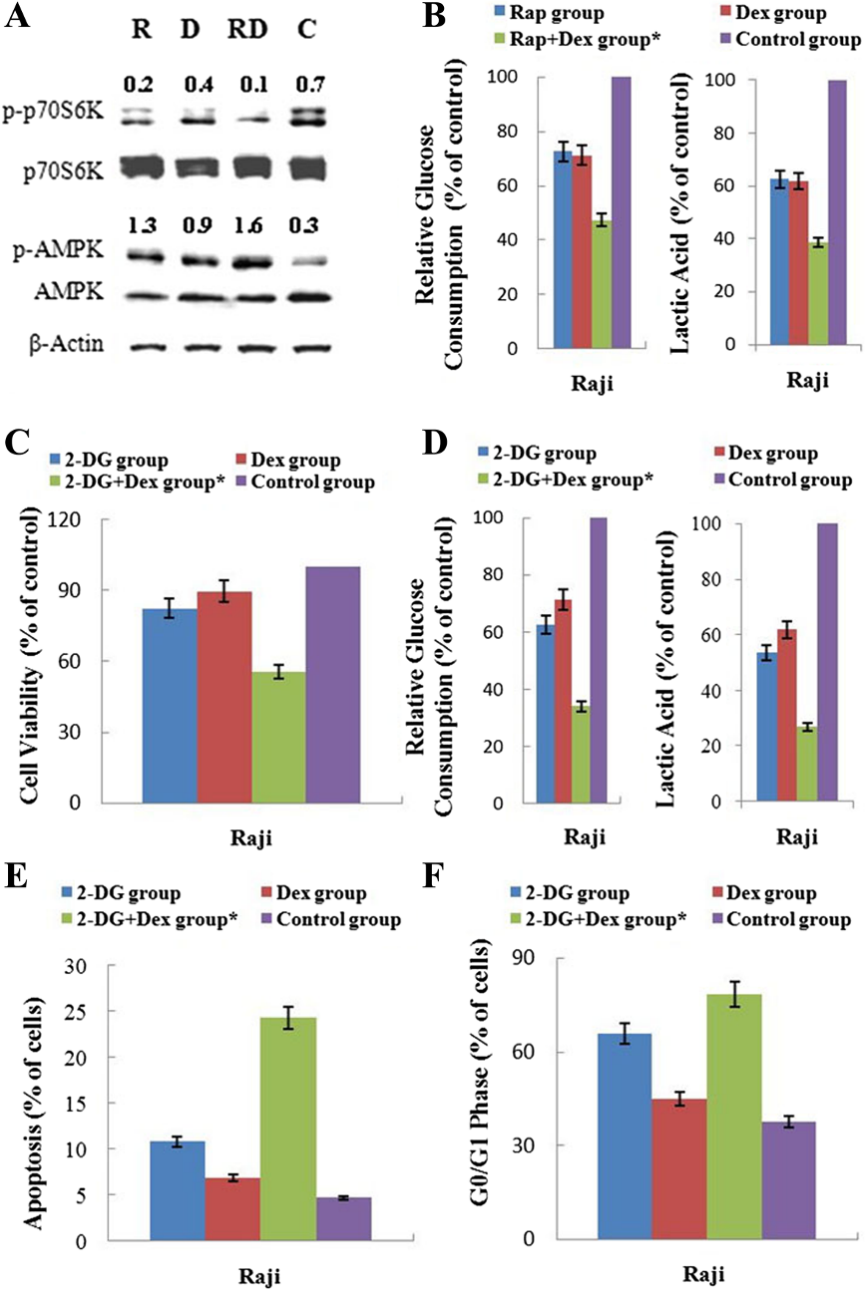
Inhibition of the p70S6K/glycolysis pathway plays an important role in Dex re-sensitization by Rap. **a** After 48 h exposure to Rap and/or Dex, cells were lysed and extracts were analyzed by western blotting for p-p70S6K and p-AMPK. Quantification is shown as a ratio of phospho-protein to total protein. **b** After 48 h exposure to Rap and/or Dex, glucose consumption and lactic acid production were measured with the Glucose (HK) Assay Kit and Lactic Acid Assay Kit, respectively. **c** After 48 h exposure to 2-DG and/or Dex, glucose consumption and lactic acid production were measured with the Glucose (HK) Assay Kit and Lactic Acid Assay Kit, respectively. **d** The viability rates of the cells were evaluated by MTT assays after 48 h exposure to 2-DG and/or Dex. **e** The early stage of apoptosis was detected by Annexin V-FLUOS/PI staining (Annexin V-FLUOS positive/PI negative) after 48 h exposure to 2-DG and/or Dex. **f** The cell cycle phases were analyzed by PI staining after 48 h exposure to 2-DG and/or Dex. *: *p* < 0.01 versus the control group, Dex group, or Rap group. R, Rap; D, Dex; RD, Rap + Dex and C, control

Previous articles have reported that p-p70S6K is a critical mediator of autophagy [25]. Rap inhibits cell growth by dephosphorylation of p70S6K and 4E-BP1 [8, 26], and dephosphorylation of 4E-BP1 is the key mechanism to reverse GC resistance [4]. We performed western blotting analysis using antibodies specific for p-p70S6K (Thr421/Ser424) and p-4E-BP1 (Thr37/46). As expected, Raji cells are null for p-4E-BP1 (data not shown), and Rap alone inhibited p-p70S6K (Fig. 6a). A stronger synergistic inhibition of p-p70S6K was detected in combined group (Fig. 6a). Our results suggest that inhibition of p-p70S6K may potentiate the cytotoxic effect of Dex.

In addition, p-p70S6K is a critical mediator of glycolytic metabolism [17]. Our data showed that along with the inhibition of p-p70S6K, Rap combined with Dex strongly inhibited the cell glucose consumption and lactic acid production (Fig. 6b). The inhibition of glycolysis leads to a decrease in intracellular ATP concentration. AMPK has been proposed as a physiological cellular energy sensor [27]. We detected the expression of AMPK phosphorylated at Thr172. Rap combined with Dex strongly induced the expression of p-AMPK (Fig. 6a). Together, these results indicate that the combination of Rap with Dex clearly inhibited p-p70S6K expression and then inhibited glycolysis in Raji cells.

To test whether inhibiting glycolysis is the core mechanism for Rap-mediated restoration of GC sensitivity, we used the glycolysis inhibitor 2-DG to replace Rap and achieved similar results. 2-DG alone inhibited glucose uptake and lactic acid production, and when combined with Dex, showed a much stronger inhibitory effect on glycolysis (Fig. 6c) and inhibited cell viability (Fig. 6d) by inducing apoptosis (Fig. 6e) and arresting the cell cycle in Raji cells (Fig. 6f).

### The combination of Rap with Dex acts synergistically on the phosphorylation of glucocorticoid receptor and dephosphorylation of ERK

**Fig. 7:**
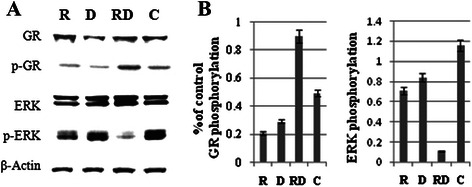
The combination of Rap with Dex acts synergistically on the phosphorylation of GR and dephosphorylation of ERK. Western blot analysis of GR, p-GR, ERK and p-ERK protein levels in Raji cells after 48 h exposure to Rap and/or Dex. Bar graphs show the ratio of phospho-protein to total protein. R, Rap; D, Dex; RD, Rap + Dex and C, control

The ability to up-regulate GR expression upon GC exposure has been demonstrated in various lymphoid leukemia cell lines and has been described as essential for GC-induced apoptosis [28]. In Raji cells, we found no obvious change in GR expression after treatment with Rap or Dex singly or in combination (Fig. 7a and b). However, p-GR at Ser211 was strongly induced by combined treatment. There was also little change in ERK but an obvious reduction of p-ERK in response to combined treatment (Fig. 7a and b).

## Discussion

Despite the good outcomes with intensive chemotherapy, GC resistance remains a major obstacle to successful treatment of lymphoblastic malignancies. Novel and less toxic treatment strategies are needed, especially for pediatric patients. Recently, the mTOR signaling pathway has received much attention as a potential target in hematological malignancies [29–31]. However, there are still some tumor cells that are resistant to Rap, for example, the Burkitt lymphoma cell line Raji. The Raji cell line possesses several Rap-resistant characteristics described by Houghton, such as the 4E-BP1-null mutation, a high level of cap-independent c-Myc expression, and the association of a4 with PP2Ac [9]. Furthermore, Raji cells are also resistant to GC. Surprisingly, the present study provides evidence that Rap combined with Dex, both at clinically achievable concentrations, interacted synergistically to inhibit Raji cell viability. This effect was found not only *in vivo* but also *in vitro*.

To unveil the underlying mechanism, we further studied the effect of the combined treatment on the cell cycle. Rap or Dex alone had no effect on the cell cycle progression of Raji cells. Combined treatment, similar to those Rap-sensitive cells, can induce G_0_/G_1_ cycle arrest in Raji cells. The down-regulation of Cyclin D1 and Cyclin A along with the up-regulation of CDK inhibitors p21 and p27 has previously been suggested to be the mechanism behind mTOR inhibitor-induced cell cycle arrest in Rap-sensitive cells [2, 32]. We achieved similar results in the combined group: a strong induction of p27, a slight up-regulation of p21, and down-regulation of Cyclin D1 and Cyclin A. Therefore, combined treatment successfully restored the sensitivity to Rap.

According to the results of the apoptosis assays, combined treatment restored the sensitivity of Raji cells to GC. Bcl-2 family members are critical regulators of the intrinsic apoptotic pathway and play critical roles in GC-induced apoptosis [23]. Members of this family can be divided into two groups: the anti-apoptotic proteins, such as Bcl-2 and Mcl-1, and the pro-apoptotic proteins, such as Bax and Bim. Published papers have verified that Rap restores GC sensitivity and induces apoptosis through the intrinsic apoptotic pathway [2–7]. Our studies showed that in Raji cells, Rap combined with Dex obviously cleaved Bcl-2 and caspase-3. Unlike the reported results [2–7], Rap and Dex alone or combined induced Bim expression clearly, and combined treatment had little effect on Bax and did not affect Mcl-1expression. The changes on bcl-2 related proteins may correlate with GC resistance in Raji cells, which need confirmation by further research. In Burkitt lymphoma cells, enhanced apoptosis in response to chemotherapeutic agents is independent of p53 and Bax [33]. ∆ψm dissipation is an early event in apoptosis activated through the mitochondrial pathway [34]. However, there are emerging data suggesting that depending on the cell system under investigation and the apoptotic stimuli used, the dissipation of Δψ_m_ may or may not be an early event in the apoptotic pathway [35]. In our study, there were no significant differences between Rap and Dex alone or combination in dissipation of ∆ψm. Further study indicated that the pan-caspase inhibitor z-VAD-fmk only partially interfered with the GC-sensitizing effect of Rap, whereas z-VAD-fmk blocked the cytotoxic effect of Dex in GC-sensitive cells [22]. The data proved that combined treatment triggers a caspase-independent cell death in Raji cells. Autophagic cell death is the most studied caspase-independent cell death [24]. Induction of autophagy-dependent necroptosis is a potential mechanism for childhood ALL cells to overcome GC resistance [22].

As Raji cells lack the expression of 4E-BP1, Rap treatment only reduced the expression of p-p70S6K and cannot arrest the cell cycle. Fortunately, dephosphorylation of p70S6K can effectively induce cell autophagy [36]. Our results reconfirmed that Rap treatment alone inhibited p-p70S6K expression and induced autophagy in 4E-BP1-null Raji cells; combining Rap with Dex increased these effects. While it is clear that autophagy is a protective mechanism at times of cellular stress, the contribution of autophagy in regulating cancer cell death or survival remains controversial [37]. In our study, the autophagy inhibitor 3-MA inhibited the viability of Raji cells in the Rap and Dex treatment alone groups. However, 3-MA did not affect the cytotoxicity of the combination treatment by inducing apoptosis. z-VAD-fmk has been reported to induce cell death via autophagy [38], which may explain why z-VAD-fmk did not fully protect the cells from the combined treatment. Our data showed that Rap combined with Dex induced cell killing depended on caspase-dependent apoptosis and caspase-independent autophagy cell death in Raji cells. Importantly, once the cytotoxicity of the combined treatment is triggered, the cancer cells will not be protected by the inhibition of apoptosis or autophagy.

How can Rap restore Dex-induced apoptosis in 4E-BP1-null Raji cells? Notably, S6K is the core regulator of glycolysis [17]. Ninety years ago, Otto Warburg [39] discovered that enhanced aerobic glycolysis distinguishes cancer from normal tissues (also known as the Warburg effect). Upregulation of the cellular metabolism (including glycolytic and oxidative phosphorylative pathways) and proliferation is an important aspect of GC resistance in ALL and may contribute to patient outcome [40]. GC resistance is directly associated with a glycolytic phenotype [41] and the activation of glycolysis has suppressive effects on the apoptotic potential [42]. The inhibition of glycolysis can reverse the GC resistance by inducing apoptosis in ALL cells [41, 43]. It is noteworthy that although Raji cells are resistant to Rap, Rap treatment alone can diminish p-p70S6K, dissipate ∆ψm and inhibit glycolysis in Raji cells. There may be a potential link among p70S6K, glycolysis and GC resistance. In support of this hypothesis, our data indicated that Rap combined with Dex clearly inhibited glycolysis, and the glycolysis inhibitor 2-DG effectively took the place of Rap. When 2-DG was combined with Dex, it recapitulated the effect of Rap combined with Dex by inducing apoptosis and arresting the cell cycle. We got the same results in Rap-sensitive T-ALL and B-ALL cell lines (data not shown).

GC resistance may be caused by a lack of GR up-regulation upon GC exposure in leukemia cell lines [44]. However, there is evidence that GC resistance in childhood ALL cannot be attributed to an inability of resistant cells to up-regulate the expression of the GR upon GC exposure, nor to differences in the GR promoter usage [45]. Another study demonstrated that the Ser211 phosphorylation site is a key regulator of GR transcriptional activation and repression [46]. Treatment with Dex results in the phosphorylation of GR at Ser211 with increased GR expression in Dex-sensitive CEM clones, whereas in Dex-resistant CEM clones, Rap + Dex elevates p-GR (Ser211) expression with no increase in GR protein [47]. Our study showed that Rap combined Dex induced the expression of p-GR (Ser211) with no increase in GR expression in Raji cells. Meanwhile, combined treatment did not influence the expression of ERK but inhibited the ERK signaling pathway by reducing p-ERK levels. Garza [5] found that the Dex-resistant cell lines have high basal levels of p-ERK relative to Dex-sensitive CEM-C7-14 cells. The p-ERK protects against GC-evoked apoptosis in sensitive T-ALL cells [47]. The inhibition of ERK also restores GC sensitivity in resistant T-ALL cells [48]. The induction of p-GR (Ser211) and reduction of p-ERK verified directly that combined treatment restored the GC sensitivity in Raji cells.

## Conclusions

Taken together, inhibition of the p70S6K/glycolysis signaling pathway plays an essential role in reversing GC resistance in Raji cells, which provides new insight into the molecular mechanisms involved in Rap reversing GC resistance. Targeting mTOR/p70S6K/glycolysis signaling pathway warrants further investigation as an attractive new therapeutic approach for GC-resistant lymphoblastic malignancies.

## Additional file

Additional file 1:
**The ARRIVE Guidelines Checklist.**

## Abbreviations

Dex: Dexamethasone
GC: Glucocorticoid
mTOR: Mammalian target of rapamycin
p70S6K: p70S6 kinase
p-p70S6K: p70S6K phosphorylation
p-4E-BP1: Phospho-4E-BP1
Rap: Rapamycin
2-DG: 2-deoxyglucose
3-MA: 3-methyladenine
4E-BP1: Eukaryotic initiation factor 4E binding protein 1
